# Empowering One Health with metagenomics

**DOI:** 10.1186/s42522-026-00225-4

**Published:** 2026-06-29

**Authors:** Erin Harvey, Kate Van Brussel, Edward C. Holmes

**Affiliations:** 1 Institute of Diagnostic Virology, Friedrich-Loeffler-Institut, Greifswald-Insel Riems, Germany; 2 School of Medical Sciences, The University of Sydney, Sydney, NSW 2006 Australia

**Keywords:** One Health, Metagenomics, Pandemic, COVID-19, Emergence, Evolution

## Abstract

In an increasingly connected world a global One Health approach to the management of human, animal and ecosystem health will be critical to effective infectious disease responses. The emergence and rapid global spread of several emerging and re-emerging pathogens in the past decade has highlighted the need for rapid, sensitive and accurate diagnostics. Metagenomics, while commonly used for research purposes for almost two decades, entered the global spotlight during the COVID-19 pandemic. In this review we discuss the impacts that metagenomic studies have had on our understanding of origins, aetiology and ecology of infectious diseases within a One Health context. We also discuss the role of metagenomics in the future of diagnostics and disease surveillance, and outline the challenges and limitations of current metagenomic methods.

## Introduction

Climate change, industrialised farming, urbanisation, human conflict and habitat loss are shaping our world into an unstable place where infectious disease emergence is increasingly commonplace [1, 2]. The One Health philosophy recognises the global interconnectedness of human, animal and ecosystem health, encouraging collaboration across disciplines. The extent to which the world is connected in the 21st century necessitates that we no longer always see disease outbreaks as a local issue to be handled by individual health services, but as a global problem that is often better addressed by consortiums of scientists, health care workers and policy makers in multiple localities, and where the links between human and animal world may be paramount.

While PCR-based approaches are usually the most rapid and cost-effective method for the detection of known pathogens, metagenomic sequencing has become a valuable diagnostic method for cases of undiagnosed and/or complex disease [3–5]. As readily demonstrated during the COVID-9 pandemic, the speed at which a novel pathogen can be identified and genomically characterised is of critical importance in epidemic and pandemic responses. Pathogen identification was an often challenging and time consuming process, with some disease agents taking years to be discovered, slowing the development of vaccines and therapeutics [6]. As a case in point, it took two years for the human immunodeficiency virus (HIV) to be identified as the causative agent of AIDS [7]. Fortunately, approaches to disease emergence and outbreak management have been revolutionised by the development and increasingly routine deployment of metagenomic sequencing. Not only does metagenomics enable the identification of new or re-emerging pathogens within hours, but because it necessarily generates genomic sequence data it also facilitates the speedy development of routine diagnostic tests (especially those based on PCR) and vaccine design. Indeed, the first mRNA vaccines for COVID-19 were designed within 48 h of the public release of the SARS-CoV-2 genome sequence [8]. More broadly, the rapid turnaround time, largely unbiased nature, and steadily decreasing cost of metagenomics has led to its widespread adoption across One Health disciplines, which will assuredly play an increasingly significant role in an uncertain future [9–14].

While the emergence of novel pathogens captures global media attention, metagenomics is now routinely deployed in increasingly diverse fields. Medical diagnostics, epidemiological and disease surveillance in animal health and agriculture, food safety, and biosecurity are just some of the areas that have witnessed the rise of metagenomic sequencing [5, 15–17]. The decreasing cost of genome sequencing, coupled with increasing data outputs and the development of powerful data analysis workflows that provide rapid and accurate results [18, 19], are enabling these techniques to be increasingly utilised. In a medical context, there is a growing uptake in the use of metagenomics for clinical diagnostics, including hospital-based, long-term metagenomic surveillance projects [5, 15], and diagnostic discoveries based largely on metagenomic sequencing are becoming increasingly commonplace and informing treatment outcomes [4, 10, 12, 20].

Herein, we provide an overview of the applications of metagenomics in the context of One Health, outlining the utility of this approach as well as some of the ongoing challenges to its deployment. While the largely unbiased nature of metagenomics allows for a wide range of applications, because of the recent high profile global outbreaks of SARS-CoV-2, mpox, hantavirus, nipahvirus and H5N1 influenza virus, we necessarily devote most attention to its applications in the context of the emergence of viral pathogens. We also discuss the changing understanding of disease aetiology as a result of metagenomic data and the broader necessity of moving from a pathogen-host understanding of microbiology to a perspective centred on ecosystem health. In addition, we outline the current limitations of metagenomics, including those reflecting global inequality, as well as its implications for our understanding of microbial diversity, ecology and evolution, and on our ability to help prevent emerging diseases in humans, plants and animals. Finally, we comment on the future possible trends in the use of metagenomics in One Health.

### Metagenomic methods and applications in One Health

**Fig. 1:**
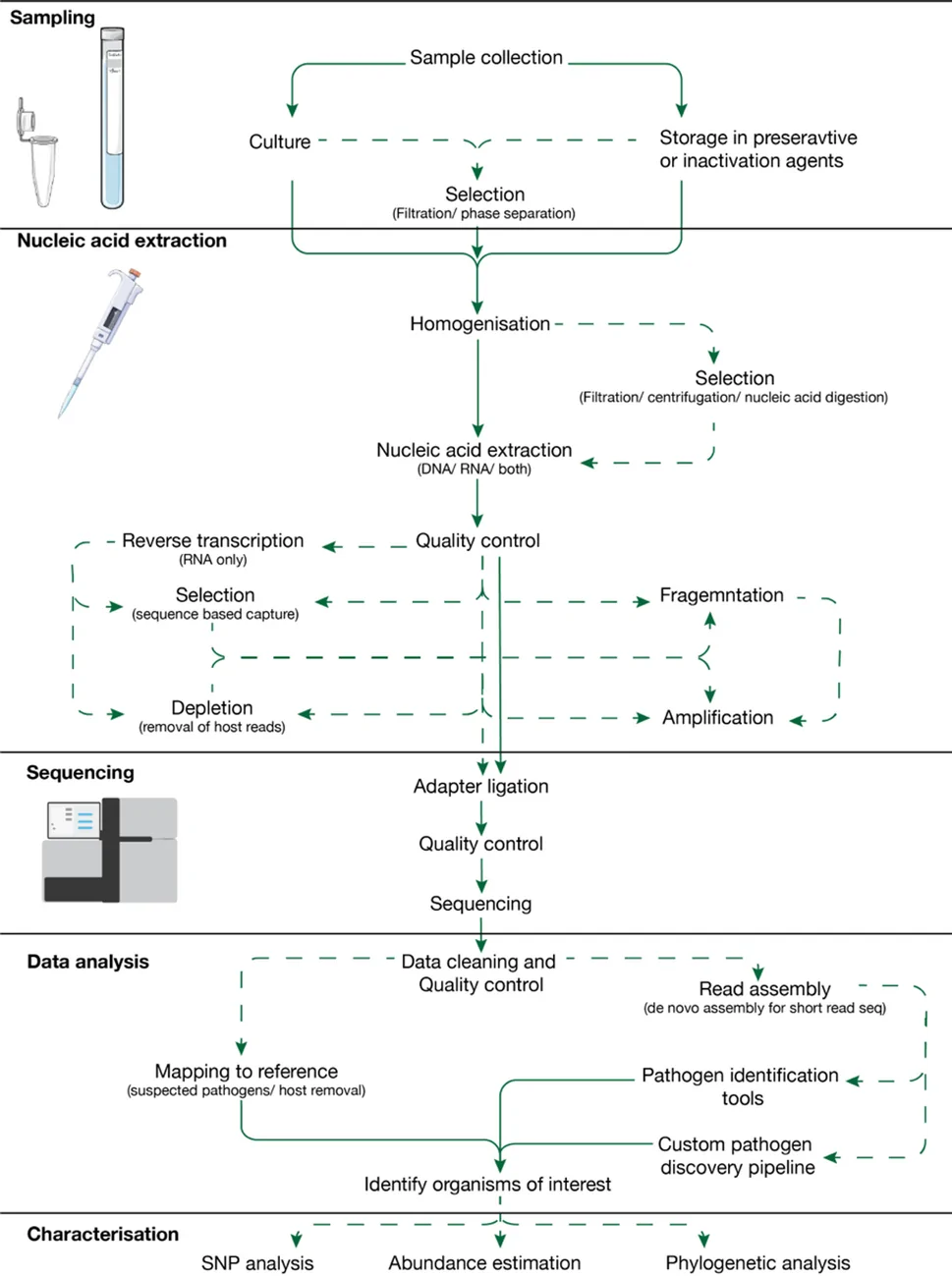
Overview of metagenomic workflows from sample collection to final data analysis. The workflow pattern is broken into five broad categories; (i) sampling, (ii) nucleic acid extraction, (iii) sequencing, (iv) data analysis and (v) characterisation. Optional steps are indicated with a broken line while steps considered mandatory are indicated with a solid line. The directionality of each step is indicated with a green arrow. Adapted from ref [24]

The term ‘metagenomics’ is commonly applied to a range of techniques and technologies depending on the field of study (Fig. 1). We define metagenomics as the high-throughput sequencing of the total nucleic acid, be it DNA and/or RNA, within a sample. A variety of methods and workflows have been developed across disciplines to accommodate various applications including tailored depletion methods, probe based selection methods and amplification methods (Fig. 1). Detailed discussions of these techniques can be found elsewhere [21–23].

Despite ongoing advances in the field, there is currently no standardised method for virus metagenomics. Unlike bacteria or eukaryotic pathogens, there is no gene that is universally conserved among both DNA and RNA viruses (reflecting their independent origins, although each has its own ‘hallmark’ set that have been used for their identification) and they exhibit enormous genetic diversity that cannot be always captured by targeted amplification or probe based systems [25]. While DNA sequencing is most often associated with the term metagenomics, DNA alone can be of limited effectiveness in pathogen detection because of the abundance of host genomic material and, more pointedly, because RNA viruses necessarily contain no DNA. On average, host DNA accounts for 94–99% of the DNA content in human respiratory samples, and methods that aim to deplete this component prior to sequencing vary in effectiveness [26]. In contrast, although more costly and logistically challenging because of RNA degradation, RNA metagenomic methods are capable of detecting all actively transcribing viruses as well as DNA-based microbes in a given sample. However, because of the over-abundance of ribosomal RNA (rRNA), which accounts for > 85% of transcripts in most transcriptomes [27], the depletion of this component (or the enrichment of any viruses present) is generally required.

Virus-specific extraction methods that include depletion of unencapsulated material or particle size selection through centrifugation or filtration can identify novel viruses at the same time reducing sequencing depth and cost [28]. Probe-based targeted enrichment methods such as TWIST and VirCapSeq can reduce the sequencing depth for virus identification [22, 23, 29] and are becoming increasingly common in studies of human populations where the pathogen responsible for a disease is likely to be from a restricted set. However, by definition these methods do not capture the entire diversity of the virome and hence may not detect a newly emerged pathogen that is not present in the target panel, or reveal the interactions among co-existing microorganisms [30]. Despite these intrinsic limitations, virus-specific extraction or enrichment methods have shown promising results when applied to waste-water and environmental sampling, identifying a range of known pathogens with as much as 20% sequence divergence from the reference genome [21]. In contrast, direct comparisons of virion-enriched metagenomics and metatranscriptomics (i.e. total RNA sequencing) with rRNA depletion have shown that while virion enrichment performed better for the detection of DNA viruses, metatranscriptomics was superior overall [31].

In theory, host genome depletion methods provide a means to capture more of the infectome and have been successfully implemented in the metatranscriptomic sequencing of many species and environmental contexts [32–35]. Host depletion could include a targeted probe-based pull-down method from the complete host genome, but more commonly refers to the depletion of rRNA, for which a number of commercial kits are available [27]. While whole host genome depletion reduces the sequencing depth required, in turn inflating the microbial component, the cost of developing a probe panel usually outweighs the benefit of a whole genome depletion and is only feasible for humans or highly studied species for which a curated genome is available. This method is often used in studies of DNA microbes, such as microbiome studies, where removal of the host genome is advantageous [36]. Alternatively, commercially available rRNA depletion kits are cost effective and functional for a range of target host species with effectiveness as high as 99.05%, although most sequence reads generated still represent host nucleic acid [37]. As an important case in point, metatranscriptomics with commercial rRNA depletion was used in the identification of (what became) SARS-CoV-2 from the bronchoalveolar lavage fluid sample taken from patient infected in Wuhan in late December 2019 [38]. Host depletion and total RNA sequencing has the added benefit in environmental samples or samples where contamination is of concern, with the analysis of read abundance helping to reveal virus-host relationships: the higher the sequence read abundance of a particular virus in a sample, the more likely it infects the host of sampling rather than being a component of host diet or microbiome, while a high number of reads for the host sampled helps to exclude the possibility of contamination. For example, the metatranscriptomic sequencing of faecal samples from wildlife and domestic animals in Australia revealed the presence of a plant pathogen classified as an exotic biosecurity threat [17]. Similarly, many virus discovery studies have utilised the huge abundance of metagenomic data produced for other purposes and freely accessible online at the Sequence Read Archive (SRA) to mine for novel virus sequences, with new bioinformatic tools being developed for this purpose [39–41].

The lack of a standardized approach in sample preparation and sequencing for virus metagenomics has been a challenge to its implementation in diagnostics, particularly as there can be major differences in protocols across sample types, host species and viruses. Human tissue or blood samples collected in sterile hospital environments where an entirely novel pathogen is the unlikely cause of disease may require a different protocol to samples of skin, faeces or gut where a complex microbiome exists. Project design for exploration purposes will also differ from diagnostic projects where a series of control samples are necessary to account for the high sensitivity of metagenomic sequencing and the abundance of viral contaminants in reagents, materials and the environment (42). Differences in available resources also dictate protocol choices. Probe based approaches and other selection or depletion methods that lead to less complex metatranscriptomes can be combined with real-time, portable sequencing technologies such as that provided by Oxford Nanopore Technologies, particularly the Minion platform which can theoretically be run in field and hospital settings producing real-time sequencing outputs [3]. These platforms also generate long sequence reads, further simplifying analysis [42]. However, because of the greater read output, short read sequencing such as that provided by Illumina platforms remain the most commonly used for virus metagenomics, particularly total RNA sequencing. While targeted probe based approaches show strong results in pathogen surveillance and diagnostics [5, 43], and provide more viral reads for analysis, it is critical that the correct probe set be employed. Commercial probes can be costly and may not be a viable option in low-resource settings where these approaches would be most beneficial due to the lack of the high performance computation resources and necessary bioinformatics expertise. Conversely, general host depletion using commercial rRNA kits incur lower sample preparation costs but require greater sequencing depth and resources for data storage, analysis and interpretation.

While metatranscriptomics enables a more detailed view of the infectome, greater sequencing depth may be required to detect low prevalence viruses, incurring greater cost and requiring greater computational resources for data storage and analysis. Indeed, the enormous amount of data generated during metatranscriptomic sequencing – which may reach the equivalent of ~ 10 human genomes per library – poses a major computational challenge, especially in resource-poor settings. High sequencing depth may be important as a viral infection can be indicated by only a small number of sequence reads if the sample is not taken during the acute stage of infection or from the infected tissue [44]. Fortunately, ultra-high throughput sequencing technology such as the Illumina Novaseq X platform can produce over 8 terrabytes of data in a single flow cell (although with considerable downsteam computational costs). Beyond constraints associated with sequencing depth, data analysis is usually not fully automated, which increases the time-component and complexity of any study. Indeed, the interpretation of results still generally requires a knowledge of virology and the principles of metagenomic analysis that extends beyond computer software alone and hence may exceed that available in most public health and veterinary diagnostic laboratories. Although research laboratories are able to use slow and resource intensive methods such as protein structure similarity analysis to detect novel viruses, clinical, veterinary or agricultural laboratories usually require rapid and cost effective analysis methods. Fortunately, advances are being made towards end-to-end metagenomic virus discovery tools through the use of cloud-based servers and the integration of artificial intelligence (AI) in detection pipelines [45]. As a case in point, publicly available web-based computational tools such as VirID and Serratus use the conserved RNA-dependent RNA polymerase (RdRp) to detect RNA viruses in metagenomic data [19, 40], but are limited by their reliance this protein alone, such that other virus genes and proteins are not considered.

More broadly, metagenomic virus discovery has developed into a major area of study in which a specialised skill set required for robust and accurate analysis, particularly the accurate identification of true from false-positive results [46]. This burgeoning field is presenting new challenges to virology and biological data management resources as virus sequence databases continue to grow by millions of entries per year. Despite this progress, the lack of a standardized approach, as well as the vast amount of data generated, continues to create challenges in the field.

### Revisiting the one pathogen, one disease ethos

Prior to the metagenomic age, viruses were generally identified through their association with overt disease and primarily in humans and anthropogenically significant plant and animal species [6]. This resulted in a narrow view of virology, in which viruses were primarily agents of disease and largely described in mammalian systems, with several viral lineages mistakenly believed to have emerged in mammals or even humans. Hepatitis delta virus (family *Kolmioviridae*) provides an illustrative example. For many years this virus was exclusively associated with human infection in the context of co-infection with hepatitis B virus (HBV). However, metagenomic studies over the last decade have found related viruses in diverse animal species and without HBV present [39, 41, 47]. Hence, through metagenomic sequencing we have begun to understand the true diversity and ubiquity of viruses, identifying whole families, orders and even phyla of novel viruses and in hosts never before studied [24, 45]. Paradoxically, however, this new understanding of virus diversity and the increasing sequencing depth has complicated the identification of the exact pathogens responsible for a particular disease as many of the viruses detected in clinical samples may not be associated with the disease state or even the host sampled. In particular, depending on the sample type, many viruses detected through metagenomic virus discovery exercises were later found to be associated with dietary, microbiome or contaminating sequences from the host microbiome, extraction reagents, collection materials or laboratory contaminants [48, 49]. For example, picobirnaviruses were originally thought to be associated with disease until their continued identification in gastrointestical samples, lack of presence in other tissues, and lack of phylogenetic clustering by host suggested that they more likely represent gut microbes [50]. Accordingly, the discovery of a microbe in a diseased host no longer necessarily means that this pathogen is the causative agent, and accurately assigning viruses to their hosts is one of biggest challenges in metagenomics [46].

Through metagenomics we are also beginning to understand the complexity of disease aetiology and the limitation of assuming that one disease is solely due to a single pathogen or that the presence of a virus or microbe is the only factor leading to overt disease. Rather, a new understanding is emerging in which disease may be the result of infection with multiple microorganisms as well as interacting factors associated with host, microbiome and environment [51–54]. An example is provided by studies of late onset sepsis, in which patients developed sepsis and organ failure following complex surgical intervention. Rather than a single pathogen, late onset sepsis was associated with changes in the gut microbiome leading to the invasion of pathogenic microbes [55]. This complex system is sometimes referred to as the ‘pathobiome’ environment [51, 54]. Similarly, it was previously believed that the lungs were a sterile environment and that respiratory disease was caused by the introduction of pathogenic viruses or microbes. However, culture-independent studies have now established that the lung is in reality populated with microbial species notoriously difficult to culture using traditional methods [53, 56]. The contribution of secondary bacterial infections following respiratory viral infection is now widely recognised, and the impact of the host respiratory and gastrointestinal microbiome as well as the host immune response are being studied for their impact on patient outcomes [57, 58]. Again, the advantage of metagenomics, particularly total RNA sequencing, is that a single data set can provide information on the virome, microbiome and host transcriptome, resulting in a uniquely detailed view on all the potential pathogens in a host and how they might interact.

In the case of human studies, comprehensive health monitoring and surveillance has made it possible to identify the causes of all infections of all types, including those where the symptoms are mild and/or many cases are asymptomatic. In contrast, in other animals, particularly wildlife, it is more likely that a disease will only be detected after a serious die-off event or significant observable disease, such that pathogens associated with mild diseases often remain unsampled [59]. In addition, while secondary infections to viral disease are frequently observed in humans and are beginning to be recognised in farmed animals, the current literature contains no examples from wildlife [60–62]. Hence, if an acute viral infection leads to a secondary bacterial infection and the death of the animal, the initial viral infection may not be detected, and even if it is detected, it may not be associated with disease. In such circumstances techniques like PepSeq that detect specific antibody responses to a diverse set of viruses (i.e., highly multiplexed serology) will likely be of benefit [63, 64], although to date they have only been applied to human populations. In addition, it is possible to use host transcriptome data to detect pathogen-specific immune responses which in turn help to identify the causative agents of disease [65, 66].

One of the major successes of metagenomic sequencing is the revelation that wildlife species serve as major reservoirs for a wide range of viral species, including potentially zoonotic viruses [67]. This work has also shown that mammalian taxa that are both speciose and often at high population density, such as some species of bats and rodents, are often particularly important reservoirs for a diverse range of potentially zoonotic viruses [68–71], as well as fur farmed carnivore species [2]. An important caveat, however, is that the detection of a virus, even within a common reservoir species and for a virus that falls within taxonomic group known to harbour pathogens, does not necessarily mean that this virus represents a zoonotic threat. For example, although many coronaviruses have been identified in bat samples, the majority have not yet been associated with host jumping events to other mammalian orders [70], may be unable to utilise human cell receptors or achieve cell exit, and may not have pathogenic properties. As a particular case in point, Cedar virus, a henipavirus harboured by Australia pteropid bats and related to the highly pathogenic Hendra and Nipah viruses, can replicate in non-bat species without causing clinical signs [72].

### Virus evolution within ecosystems

**Fig. 2:**
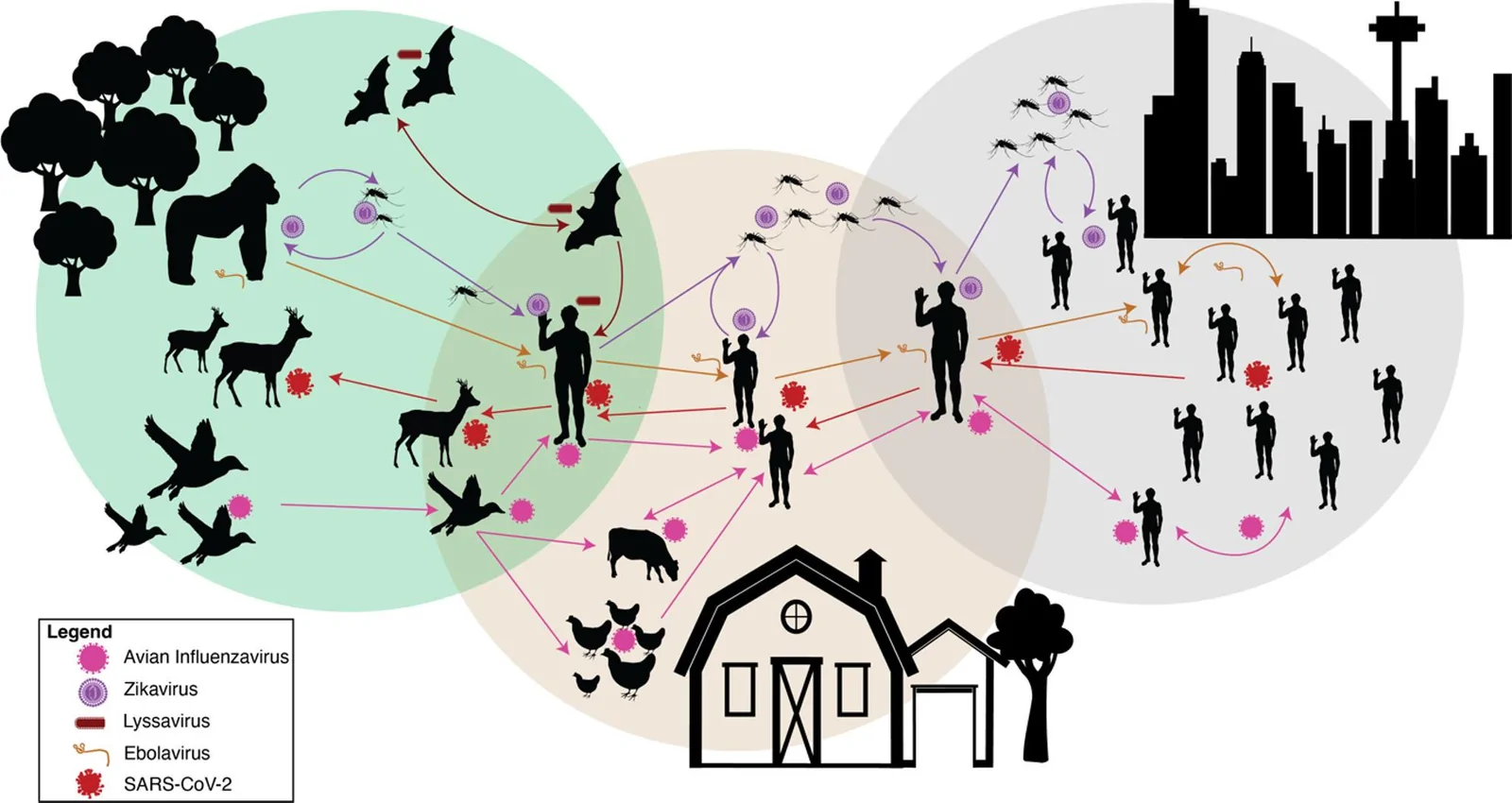
Virus movement from wild to peri-urban and urban settings, with the latter having the potential to seed epidemics and pandemics. The green sphere indicates the wild environment, the brown sphere indicates the peri-urban environment, and the grey sphere indicates the urban environment. Animal silhouettes are indicative of host order, not any specific species. Arrows indicate the direction of transmission

Although emerging viruses have been a major research topic in recent years, a fuller understanding of this process, particularly within a One Health framework, requires that humans are not always viewed as the end-point in studies of disease emergence and instead focuses on how viruses flow through interconnected ecosystems. Indeed, much of the language used in virus discovery is centred around human disease, with terms like ‘zoonosis’ and ‘spill-over’ commonly used and which humans are the last link in a chain of emergence. A zoonosis refers to events in which a pathogen is transmitted from animals into humans, while in common parlance spillover refers to the initial transmission of a pathogen from one species to a new host, often without onward transmission within that new host, which is again usually a human. For example, each case of human rabies is a dead-end spillover infection from animals (particularly dogs) with no onward transmission, as also appears true of other lyssaviruses (Fig. 2) [73]. In contrast, cross-species transmission (or host-jumping) events better describe the spillover and establishment, via onward transmission, of a pathogen in a new host, and have no necessity to be human-focused (Fig. 2).

While some viruses are strongly host specific, reflecting long-term virus-host associations and only occasional cross-species transmission, others appear to be host generalists, infecting diverse species (including, for example, multiple mammalian orders), and hence considered to be a greater risk of zoonotic emergence [2, 67]. For example, influenza virus frequently spills over from avian reservoirs, infecting a variety of mammalian species including humans. Although most spillovers of avian influenza virus to humans result in dead-end infections with no onward transmission, occasionally the virus establishes itself in mammalian hosts. A recent example is provided by the high profile outbreak of H5N1 highly pathogenic avian influenza virus in multiple mammalian species including cattle, facilitating the evolution of some of the mammal-adapting mutations that may eventually enable successful human emergence [74] (Fig. 2). In the cases of influenza and SARS-CoV-2 there is also evidence of ‘spill-back’ or ‘reverse zoonosis’, in which the virus that has emerged in humans then jumps from humans into domestic animals or wildlife, including the transmission of multiple variants of SARS-CoV-2 from humans to white tailed deer in the USA [75] (Fig. 2). This again highlights why it is necessary to move away from a human-focused view of disease emergence. Indeed, while a multitude of virus discovery studies have been directed toward identifying potential zoonotic viruses, few have attempted to understand virus emergence and evolution from the perspective of the total ecosystem. Such studies could be advanced through the metagenomic sequencing of all the animal species that interact within a particular environment rather than that just those commonly thought to be viral reservoir [76].

While humans can equally receive and contribute to virus host jumping events, we are clearly the major contributors to habitat disruption and changes in host species interactions. Indeed, the movement of viruses from natural habitats to peri-urban and urban environments is perhaps the biggest predictor of pandemic viral emergence events (Fig. 2) [77–79]. These events are commonly driven by human activities. For example, outbreaks of Ebola virus in West Africa are linked to regional political conflicts and the displacement of people, as well as deforestation and the encroachment of human peri-urban settlements on natural habitats. The 2014–2016 outbreak of the virus was associated with consumption of bushmeat in a peri-urban settlement, and escalated through the movement of infected people from the small village to local urban settlements followed by onward transmission to major urban centres [80]. Similarly, deforestation and habitat disruption are likely drivers of the 2023–2025 reemergence of Oropouche virus in Brazil and surrounding countries [81]. Human mediated changes in biodiversity have also had a major impact on disease emergence and re-emergence. As a case in point, the introduction of the mosquito species *Aedes aegypti* through the movement of people during the slave trade from Africa to South America has had devastating effects in the latter region [82], particularly as it is the vector of many devastating viral diseases including Chikungunya, Yellow fever and Zika. An outbreak of Zika virus in Brazil in 2015 led to between 497,593 and 1,482,701 cases according to estimates from the Brazilian Ministry of Health, and the virus becoming endemic on the continent is suspected to have occurred as the result of human travel between French Polynesia and Brazil [83].

**Fig. 3:**
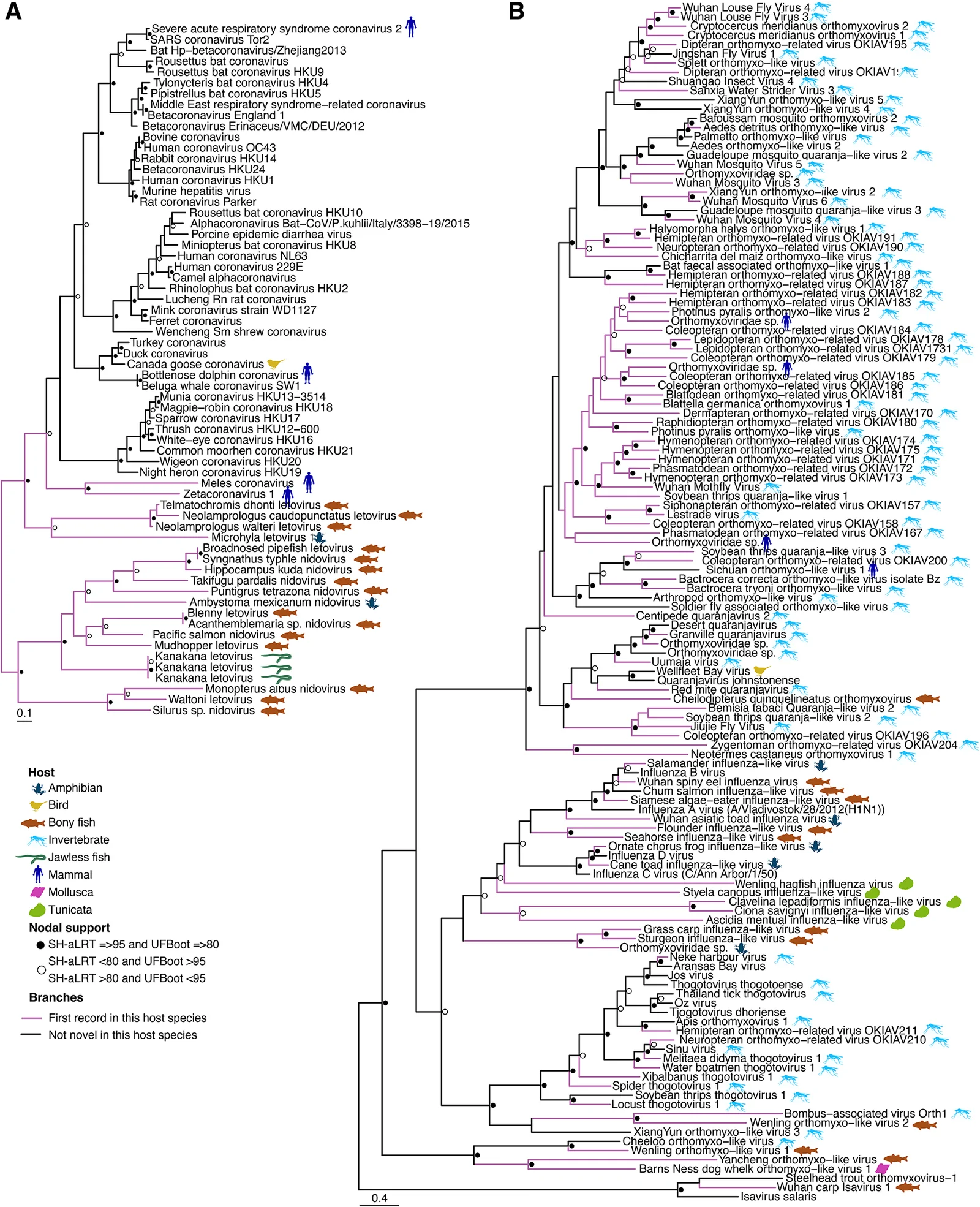
Phylogenies of the families (**A**) *Coronaviridae* (subfamily *Orthocoronavirinae*) and (**B**) *Orthomyxoviridae* as illustrations of the diversity of RNA viruses. Virus sequences discovered using metagenomic have a coloured animal symbol representing their host species. Any virus sequence discovered by metagenomic sequencing that represents the first ever occurrence of viruses from this family in the given host are highlighted by the purple branches. Maximum likelihood trees of the RdRp sequence were estimated using IQ-TREE 2 [90]. Nodal support was tested using the SH-like approximate likelihood ratio test and ultrafast bootstraps [91]. Both trees were midpoint rooted for clarity and the scale bars depict the number of amino acid substitutions per site

Metagenomic virus discovery has similarly revolutionised our understanding of virus diversity and evolution, as well as the role played by viruses in the global ecosystem. Viruses likely infect every living organism on earth, including viruses themselves, and may play a larger role than merely acting as pathogens. Increased sampling has led to the expansion of the family *Coronaviridae* to include a diverse range of hosts including bony fish [84] and jawless vertebrates such as the hagfish [85], but also to novel viral genera (with unusual genomic features such as segmentation) in carnivore species in Europe and Asia, in turn emphasising how poorly some taxa have been sampled [86, 87] (Fig. 3a). Indeed, whole new orders of viruses have been uncovered through metagenomic sequencing. An important example are the *Jingchuvirales* – an order of negative-sense RNA viruses with a wide variety of genome structures [88]. These viruses were initially identified in studies of invertebrates and assumed to be invertebrate specific, but more recent studies have shown that these viruses also infect vertebrates, including several independent mammalian lineages, and have established endogenous infections suggesting a long host-virus evolutionary history [41, 68, 89].

The discovery of such an extensive and diverse virosphere has necessitated a refocus of studies of virus evolution away from simply identifying novel viruses and toward understanding the processes behind host specificity and cross-species transmission. Identifying viruses that have the ability to jump host barriers and achieve sustained transmission in their new host is a key goal, informing the growing number of studies in ‘zoonotic risk assessment’ that aim to identify those viruses with the greatest chance of emerging in human populations [92, 93]. Importantly, the large scale metagenomic studies of particular ecosystems can reveal the species involved in transmission networks and from this the genomic determinants of viral ‘generalists’ that are seemingly better able to jump between diverse hosts [76, 87]. Although ecosystems-based approaches to understanding virus host range and zoonotic potential are likely the future of pandemic prediction, this requires the research effort of large consortia including clinicians, veterinarians, ecologists and infectious disease specialists to create meaningful output. Clearly, such an approach will not be viable without large-scale and long-term funding that is often not achievable in the regions where disease emergence is of greatest concern.

Another advantage of metagenomics in this context is that if present at sufficient abundance it may be possible to sequence complete viral genomes, in turn providing greater phylogenetic resolution which can aid researchers in tracking the source of outbreaks as they occur [12]. In particular, the ability to generate and analyse genomic data enables researchers to better understand the potential source of transmission and whether an outbreak is the result of a single zoonotic transmission event or multiple such events [10, 12]. Importantly, depending on the virus species and time and location of sampling, retrieving a full genome may not be possible, although the sequences obtained can be used to design targeted sequencing approaches.

### Metagenomics in global pathogen surveillance

A key principle in One Health is the necessity to remove geopolitical barriers in research and create a global community of knowledge sharing. The importance of this was never more evident than in the early days of the COVID-19 pandemic, when researchers in different countries collaborated to identify and characterise the virus and assess the risk to the public and design tests and vaccines against the virus. Early in the genomic era it was recognised that infrastructure and defined principles were required to enable and encourage data sharing throughout the global research community [94]. Open access platforms such as NCBI (National Centre for Biotechnology Information) and ENA (European Nucleotide Archive) provide infrastructure for the sharing of both annotated genomes and raw sequencing data (such as the SRA also administered by the NCBI). In the wake of the COVID-19 pandemic many millions of viral genomes were sequenced and the global sharing of these data was a key pillar of the global pandemic response [95]. Metagenomic virology undoubtedly hinges on free and equitable availability of this data. Encouragingly, since the COVID-19 pandemic new platforms for the sharing of genome sequence data have been developed, including community led initiatives such as Pathoplexus (https://pathoplexus.org/). However, it is also clear that these efforts need to extend beyond human pathogens to veterinary viruses. As such, highly curated data sharing platforms are essential as metagenomic sequencing increases. This will require a global effort to support the hosting and maintenance of databases that are not owned by any one country or political entity, thereby ensuring free and equitable access to data download and upload.

**Fig. 4:**
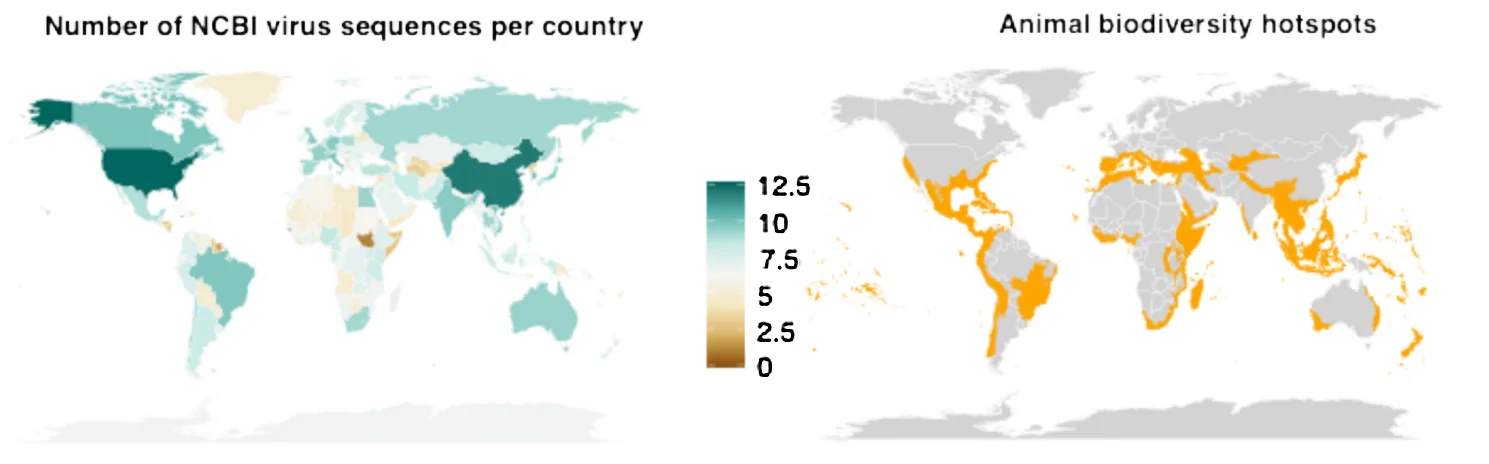
World maps illustrating virus sequence output per country relative to animal biodiversity hotspots. The shape.file was obtained from [100]. The R script map_data(‘world’) was used to get the map, while the scripts ggplot, dplyr and ggpubr were used to make the maps. The map on the left shows that wealthier countries with more resources (e.g. USA and China) sequence more viruses than under resourced countries (such as counties in Africa and South America). The map on the right shows that these under resourced countries contain large animal biodiversity hotspots, yet do not sequence viruses to the depth of wealthier countries. Virus sequences belonging to families that infect plants, bacteria and protists were excluded from the data used to generate this figure

There is increasing interest in large scale metagenomic surveillance studies both in clinical and environmental settings [96–98]. While the cost of metagenomic sequencing has declined dramatically in the past decade, there are still obvious biases in our study of the global virome, particularly towards regions with greater availability of funding and research infrastructure (such as Western Europe, North America and China) that do not always align with the world’s richest animal biodiversity hotspots (Fig. 4). The global reliance on PCR testing and high throughput sequencing for the detection of emerging variants did lead to the establishment and dramatic improvement of sequencing facilities across regions previously unable to access these services. In 2018, only seven African countries had public health sequencing capacity, yet in the years following the COVID-19 pandemic more than 38 countries on the continent now have sequencing capacity [95]. Despite these advances, supply chain challenges, lack of funding, and shortages in trained personnel still limit metagenomic capabilities in low and middle income regions. Providing equitable resourcing, training and funding is key to achieving the cross boarder surveillance necessary to meaningfully apply a one health framework to disease emergence. NGOs and international research institutions such as the Wellcome Trust and Institute Pasteur have dedicated pathogen genome sequencing efforts [99], and we recommend that virus metagenomics be included by providing funded courses and targeted calls for project based funding.

Political instability also threatens implementation of the One Health framework for surveillance and pathogen discovery. The One Health philosophy relies on principles of equality and the free sharing of resources, knowledge and information across borders and cultures. One Health initiatives often rely heavily on non-government organisations, open access publications and data sharing systems and international aid programs [101]. NGOs such as the World Health Organisation (WHO) strive to facilitate international pandemic preparedness efforts by holding intergovernmental working groups. Most recently the WHO Pandemic Agreement acknowledged the importance of data sharing in pandemic prevention and preparedness and in response established the Pathogen Access and Benefits Sharing (PABS) system for the rapid and timely sharing of information related to potential pandemic outbreaks, although it is unclear how this will work in practice. There are clear links between political instability and human conflict to disease emergence [1]. In addition, the global swing towards right wing populist governments has seeded distrust in the medical and research communities, with wide ranging impacts on vaccination rates, disease surveillance, freedom of information and funding availability both for research and disease control measures [102, 103]. That these threats to the global management of research and disease surveillance and control measures are occurring at a time when we are experiencing increasing instances of disease emergence is concerning. One current focus is the spread of highly pathogenic H5N1 avian influenza virus, which is having global impacts on wildlife conservation and agri-businesses [104, 105]. Host-jumping events of H5N1 into mammals are now well documented, first in cats and then in marine mammals, and arguably of more concern in farmed cattle [74, 106, 107]. Globally, the emergence of this virus is contributing to already strained global food supply issues [108].

It is increasingly clear that pathogen (re)emergence is a global issue which must be tackled by large scale consortia across multiple disciplines and without any one government or entity controlling research outputs. While efforts largely remain focused on zoonotic events rather than the complex movement of viruses through ecosystems and the impacts of rapid change on these processes, we limit our research to reactive intervention and speculation rather than real meaningful prediction and preparedness. As global interconnectedness increases and international outbreaks become more commonplace, understanding the processes behind the ability of certain virus species to readily jump hosts has never been more relevant.

## Conclusions

Metagenomics has revolutionised our knowledge of the biodiversity of the microbial world. Our understanding of microbial diversity and ecology has been greatly enhanced by the ability to characterise microbes and viruses beyond those associated with overt disease or detectable in laboratory culture and diagnostic techniques. Hence, rather than viewing infectious disease as involving ‘one pathogen – one disease’, we can now see it as a complex ecosystem or pathobiome. The decreasing cost and increasing accessibility of metagenomic technologies, combined with a One Health philosophy of sharing of open data, bioinformatic tools and information has created a global community within the field of metagenomics. However, a strong geographical bias still exists, disadvantaging researchers in low-recourse regions. The sharing of skills and information globally is critical to a rapid and efficient pandemic response in the case of future emergence events, not only in human disease but also for agriculture and wildlife. The global research effort is being increasingly threatened by government intervention restricting the sharing of data and controlling research outputs. It is critical that in the face of a rapidly changing world, impacted by climate change, severe weather events, urbanisation and habitat loss, that we consider the role of the global ecosystem in human and environmental health. Finally, it is essential that we remove the geographical bias in metagenomic research and create an equitable research environment through international aid, NGOs, investment in health and diagnostic systems, as well as international training programs to facilitate rapid and effective global disease surveillance networks.

## Data Availability

No datasets were generated or analysed during the current study.
